# The Effect of Exogenous Melatonin on the Photosynthetic Characteristics of *Rhododendron simsii* Under Cadmium Stress

**DOI:** 10.3390/plants14010125

**Published:** 2025-01-03

**Authors:** Haochen Di, Ying Liang, Yuting Gong, Songheng Jin, Yanxia Xu

**Affiliations:** 1 Jiyang College, Zhejiang A&F University, Zhuji 311800, China; dhc200528@163.com (H.D.); liangy202303@163.com (Y.L.); 15052297296@163.com (Y.G.); 2 The Nurturing Station for the State Key Laboratory of Subtropical Silviculture and School of Forestry and Biotechnology, Zhejiang A&F University, Hangzhou 311300, China

**Keywords:** heavy metal, *R. simsii*, MT, prompt fluorescence, 820 nm transmission kinetics, delayed fluorescence, JIP test, photosynthesis

## Abstract

*Rhododendron simsii* (*R. simsii*), a significant ornamental plant species, is adversely affected by the severe soil heavy metal pollution resulting from rapid industrialization, particularly in terms of its growth environment. Cadmium (Cd), a representative heavy metal pollutant, poses a significant threat to plant growth and photosynthetic physiology. Despite the importance of understanding Cd stress resistance in rhododendrons, research in this area is limited. This study focused on the role of exogenous melatonin (MT) in mitigating Cd-induced stress, emphasizing its impact on photosynthetic physiology. Gas exchange parameters, prompt and delayed fluorescence (DF), 820 nm modulated reflectance (Mr_820_), and antioxidant enzyme activity, were measured. The findings revealed that under Cd stress, MT-free treatment imposed a more severe limitation on both stomatal and non-stomatal processes in *R. simsii* leaves, significantly reducing the net photosynthetic rate. In contrast, exogenous MT improved photosynthetic efficiency by increasing the maximum photochemical efficiency of photosystem II, the quantum yield of electron transport, and the photosynthetic performance index. DF and Mr_820_ analysis demonstrated that MT provided robust protection to both the donor and receptor sides of photosystems I and II. Furthermore, MT significantly decreased malondialdehyde (MDA) content, a marker of oxidative stress, and enhanced the activity of antioxidant enzymes, including superoxide dismutase (SOD) and guaiacol peroxidase (POD). In conclusion, exogenous MT plays a critical role in alleviating Cd-induced stress by enhancing antioxidant defense mechanisms and safeguarding the photosynthetic apparatus, thereby improving the Cd tolerance of *R. simsii.*

## 1. Introduction

Cadmium (Cd), a considerably hazardous pollutant, being both highly mobile and toxic, is always a major environmental and public health risk due to its prevalence in soil. The geometric mean concentration of Cd in the agricultural soils of China was found to be 0.473 mg·kg⁻^1^ in a 2022 study, which is 1.58 times higher than the risk screening value for Cd in farmland [1]. It shows that a lot of farmland in China does not meet safety standards. This situation refers to severity in environmental contamination in places such as the Yunnan Province, the Guangxi Zhuang Autonomous Region, the Hunan Province, the Guangdong Province, and the Fujian Province. However, due to the high solubility and mobility of Cd, it can easily spread from the contamination source that cleans other parts of the soil, reaching the water bodies and agricultural products surrounding it. Through absorption from the roots, plants take up Cd, which is then stored in the roots, leaves, and seeds. When such plants are eaten, Cd is put into the food web, where the crucial risk of long-term exposure is related to some severe health disorders, such as renal dysfunction, skeletal disease, and carcinogenicity [2].

Cd toxicity creates a complex range of responses like morphological change, physiological imbalance, biochemical dysfunction, and ultrastructure modification in plants [3]. Upon entry through the root system, Cd aggravated the oxidative stress by stimulating the formation of excessive amounts of reactive oxygen species (ROS) [4,5]. H^⁺^-ATPase inactivation destroys the integrity of the plasma membrane, which, in return, results in increased membrane permeability, lipid peroxidation, and cell disintegration [6]. Plants further develop antioxidant defense mechanisms, utilizing the enzymes superoxide dismutase (SOD), peroxidose (POD), catalase (CAT), and ascorbate peroxidase (APX) to degrade the excess ROS and ameliorate the oxidative stress [7].

Furthermore, Cd disturbs the formation of the leading minerals’ transport and uptake channel, which are important for the physiological functions of major minerals like iron, calcium, and manganese [8]. For example, Cd significantly inhibits Fe3+ reductase activity in roots, thus reducing the extent of Fe^2+^ conversion, which is the main form of iron absorption via the roots of higher plants, resulting in obvious iron deficiency in plants leads [9]. Iron deficiency disrupts the synthesis of porphyrin rings, key precursors for chlorophyll production, ultimately reducing the levels of photosynthetic pigments [10]. In the molecular aspect, Cd exposure may affect the structural integrity and functional activity of DNA and cause a change in metabolic pathways [11]. Physiologically speaking, one of the earliest plant responses to Cd stress is stomatal closure [12], which restricts transpiration, but since chlorophyll metabolism is interlinked with transpiration, this restricts photosynthesis too [13]. The primary impacts on the photosynthetic system include the inhibition of chlorophyll synthesis, structural and functional damage to photosynthetic organs, a reduction in photosynthetic efficiency, and the cracking of the hydraulic continuity of the electron transport chain to the ATP synthesis, which in turn inhibits photosynthetic carbon fixation.

Melatonin (MT), a plant-derived indole compound and a natural component of plants, is implicated in plant metabolic regulation [14]. As a signaling molecule, MT is in charge of coordinating a number of functions, including circadian rhythm homeostasis, the germination of seeds, the growth of roots and flowers, and a variety of physiological responses to environmental stresses [15]. Given its role as a major antioxidant, MT scavenges ROS and is thus crucial in imparting cellular protection against oxidative damage [16]. Moreover, through molecular binding, MT is able to regulate the indicium of pivotal phytohormones, such as auxin (IAA), gibberellin (GA), abscisic acid (ABA), and ethylene (ET), allowing for the formation of an intricate signaling system, and together they impact plant growth, development, and abiotic stress resilience [17].

MT’s role as an endogenous signaling molecule has been shown to mitigate adverse effects on photosynthesis under stress conditions. For instance, studies on tomatoes have demonstrated that MT application significantly enhances the net photosynthetic rate (Pn), the maximum quantum efficiency of PSII (Fv/Fm), and the total chlorophyll content, effectively alleviating the reduction in photosynthetic efficiency induced by salt stress [18]. Additionally, MT may influence stomatal behavior via the MT receptor CAND2/PMTR1, thereby modulating the photosynthetic carbon cycle through the regulation of sugar metabolism, gluconeogenic pathways, and the dynamics of transient starch degradation and transport [19].

*Rhododendron simsii* (*R. simsii*), a species of significant horticultural importance in China, is known for its aesthetic and medicinal properties [20]. This species is predominantly adapted to acidic high-altitude soils and exhibits sensitivity to saline conditions. Under abiotic stress, particularly elevated temperatures, *R. simsii* enhances its antioxidant defense mechanisms by increasing the activity of antioxidant enzymes and the content of proline, thereby reducing the damage caused by ROS [21]. Our previous study suggests that MT can minimize the damage to Rubisco caused by heat stress by increasing RhRbsA expression levels, while also promoting ATP production under heat stress by up-regulating the expression of RhATPB [22]. Although *R. simsii* demonstrates tolerance and accumulation capacity for heavy metals such as cadmium (Cd), copper (Cu), and zinc (Zn), the specific mechanisms underlying its resistance to Cd, especially in the context of its extensive horticultural applications, remain largely unexplored. Planting *R. simsii* in areas heavily contaminated with Cd not only helps improve soil quality and rehabilitate the ecosystem, but also generates sustainable economic benefits, enhances biodiversity, and raises the public awareness of environmental issues. Its multifaceted positive impact makes this species a promising tool for environmental management. Consequently, elucidating the adaptive mechanisms of *R. simsii* to Cd-contaminated environments has become a focal point of research.

Current academic research on MT-induced Cd tolerance is predominantly concentrated on agricultural crops [23,24,25]. For example, a research team from Zhejiang University delved into the role of MT in alleviating Cd stress and detoxification in tomatoes [23]. Their investigation unveiled that MT augments Cd uptake and assimilation by modulating the expression of pertinent genes and activating specific enzymatic pathways. Under Cd stress, MT also stimulates the biosynthesis of downstream sulfur-containing metabolites, including cysteine, glutathione (GSH), and phytochelatins (PCs), which are pivotal in regulating Cd tolerance through the modulation of redox homeostasis. In research studies on cotton [24], it was found that the external application of MT helps alleviate Cd stress by delaying root senescence, promoting root development, and regulating Cd transport. Moreover, the synergistic effect of ZnO-NPs and MT can enhance the Cd tolerance of kale by regulating its physiological and biochemical processes [25]. However, there is a significant lack of research on the effects of melatonin on cadmium stress in ornamental plants.

In this study, we evaluated the effects of exogenous MT on Cd-stressed *R. simsii* seedlings, particularly focusing on photosynthetic performance. Through comprehensive analysis of gas exchange parameters, chlorophyll fluorescence, delayed fluorescence (DF), and 820 nm modulated reflectance (Mr_820_) in *R. simsii* leaves subjected to Cd and CdMT treatments, we aim to provide an in-depth understanding of the molecular mechanisms involved in plant responses to heavy metal stress and a scientific basis for utilizing MT as a potential plant growth regulator.

## 2. Results

### 2.1. Phenotypic Changes in R. simsii Under Exogenous MT and Cd Treatments

**Figure 1:**
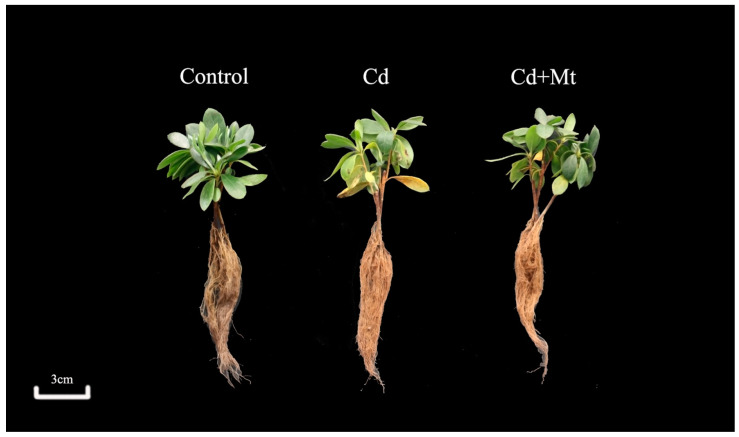
Effects of MT on the phenotype of *R. simsii* under Cd treatment after 10 days.

Significant variations in growth were observed among *R. simsii* seedlings across different treatment groups over the 10-day experimental period (Figure 1). In the control treatment, seedlings exhibited healthy growth with full glossy leaves, indicating that external factors did not adversely affect seedling development. In contrast, seedlings exposed to Cd treatment displayed marked growth inhibition, with leaves appearing wilted and yellowed. Furthermore, some leaves showed signs of abscission, and leaf tissues near the root zone displayed ulcerative damage, highlighting the adverse effects of Cd stress on plant vitality. In the CdMT treatment, the growth condition of *R. simsii* seedlings was intermediate between the control and Cd treatments. Although some yellowing and leaf wilting were still alive, the overall health and appearance of the leaves showed improvement compared to the Cd treatment.

**Figure A1:**
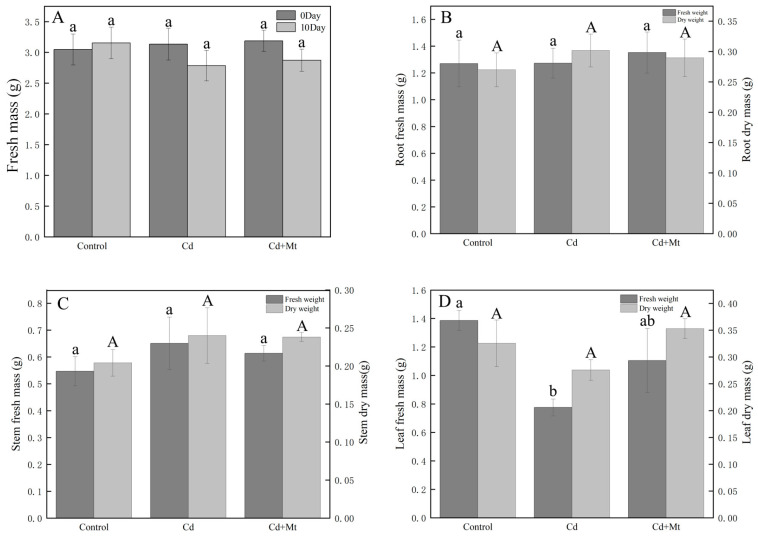
The effect of exogenous MT on fresh weight and dry weight in R. simsii under cadmium stress after 10 days. (**A**) Plant fresh and dry mass; (**B**) root fresh and dry mass; (**C**) stem fresh and dry mass; (**D**) leaf fresh and dry mass. Different letters indicate significant differences determined using Duncan’s multiple range test at *p* ≤ 0.05. Three seedlings were combined and treated as a single sample. Data are presented as the mean ± SE (n = 6; independent biological replications).

When comparing the overall fresh weight of the plants (Figure A1), the Cd treatment group at day 10 showed a slight reduction in fresh weight compared to the control group, while the fresh weight level of the CdMT treatment group was somewhere between the two. When measuring the fresh weight of different plant parts, the Cd treatment group exhibited a significant decrease in the leaf fresh weight, while the CdMT treatment group showed a relatively smaller reduction in the leaf fresh weight. This suggests that the application of exogenous MT provided some protective effects on the leaf tissue of the plants.

### 2.2. Gas Exchange

**Figure 2:**
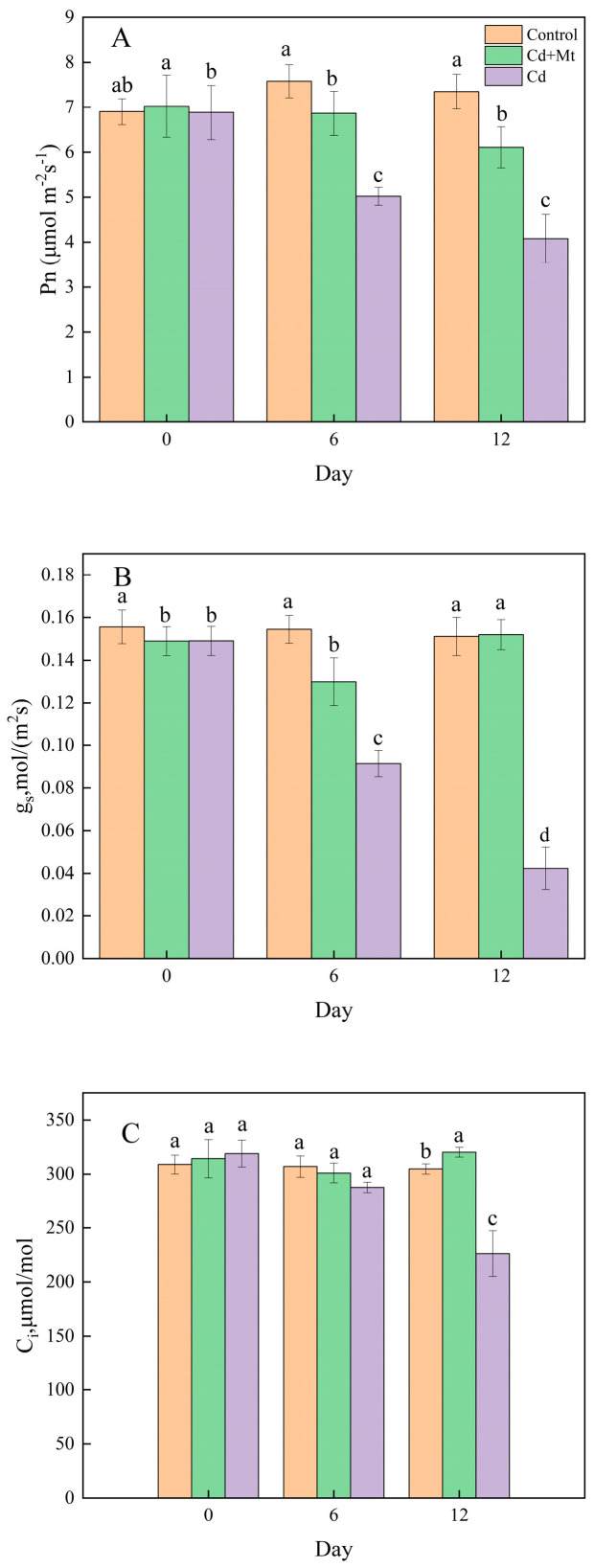
The effect of exogenous MT on gas exchange in *R. simsii* leaves under cadmium stress. (**A**) Net photosynthetic rate (Pn); (**B**) stomatal conductance (Gs); (**C**) intercellular CO_2_ concentration (Ci). Each value is the mean ± SE (n = 3). Different lowercase letters indicate a significant difference at *p* < 0.05.

The gas exchange process in plant leaves is fundamental to plant physiology, influencing growth, environmental adaptation, and ecosystem functionality. As illustrated in Figure 2A, with prolonged treatment time, the net photosynthetic rate (Pn) of *R. simsii* leaves in both the CdMT and Cd treatment groups showed a sustained decrease relative to the control treatment group. By the 12th day, Pn values in the CdMT and Cd treatments were reduced by 13.0% and 40.7%, respectively, highlighting a substantially greater down-regulation in the Cd treatment group. Furthermore, Figure 2B demonstrates that stomatal conductance (Gs) in the Cd treatment group declined sharply over time, showing reductions of 36.7% and 71.6% on the 6th and 12th days, respectively. In contrast, the CdMT treatment exhibited a nonlinear response, with an initial decrease followed by partial recovery; while Gs was significantly reduced by day 6, it recovered to levels nearly comparable to the control by day 12. This pattern aligns with intercellular carbon dioxide concentration (Ci) trends shown in Figure 2C, where Ci dynamics mirrored those of stomatal conductance across treatments. These findings suggest that exogenous MT application in the CdMT treatment mitigated Cd-induced stress, resulting in less severe physiological damage to the leaves compared to the Cd-only treatment. This protective effect of MT is evident through the improved maintenance of gas exchange parameters, especially in the later stages of exposure.

### 2.3. Prompt Chlorophyll Fluorescence

**Figure 3:**
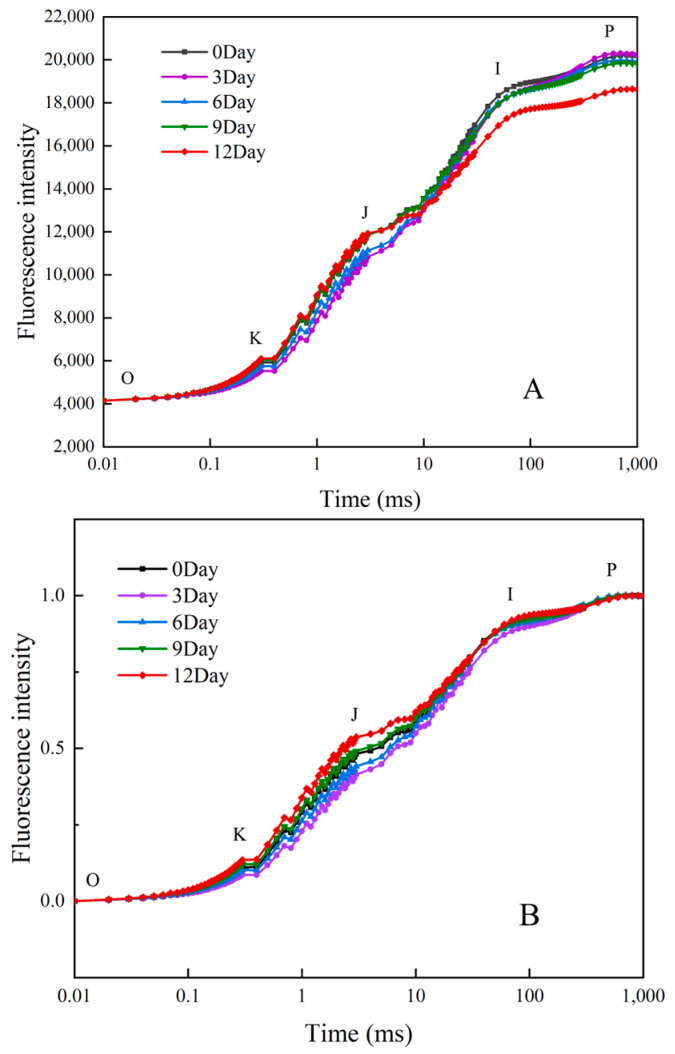
Chlorophyll fluorescence kinetics curves (**A**) and standardized chlorophyll fluorescence curves (**B**) for *R. simsii* leaves under cadmium stress. The letters O, K, J, I and P refer to the selected time points used by the JIP test for the calculation of structural and functional parameters.

During the Cd stress treatment of *R. simsii* leaves, the activity of PSII was assessed via transient fluorescence analysis. As shown in Figure 3A, the chlorophyll fluorescence (OJIP) curve exhibited typical multiphase changes, illustrating the dynamic behavior of the electron transport chain within PSII. Over the course of Cd treatment, the O-J phase displayed a decrease followed by an increase, while the I-P phase exhibited an initial increase that subsequently decreased. By the 12th day, fluorescence intensity at the P point had declined by 7.81%, indicating a reduction in the number of active PSII reaction centers. To address variability in the initial and terminal points of the OJIP curves, these curves were normalized, as depicted in Figure 3B. This normalization revealed that J-point fluorescence intensity initially decreased with Cd exposure, approximated baseline levels midway through the Cd treatment, and then showed a marked increase by day 12. Additionally, as the Cd stress duration increased, a distinct K band emerged on the OJIP curve, transforming it into an OKJIP pattern, likely signaling damage to the donor side of PSII. This progression suggests that prolonged Cd stress detrimentally affects the functional integrity of PSII, particularly impacting electron donation processes.

**Figure 4:**
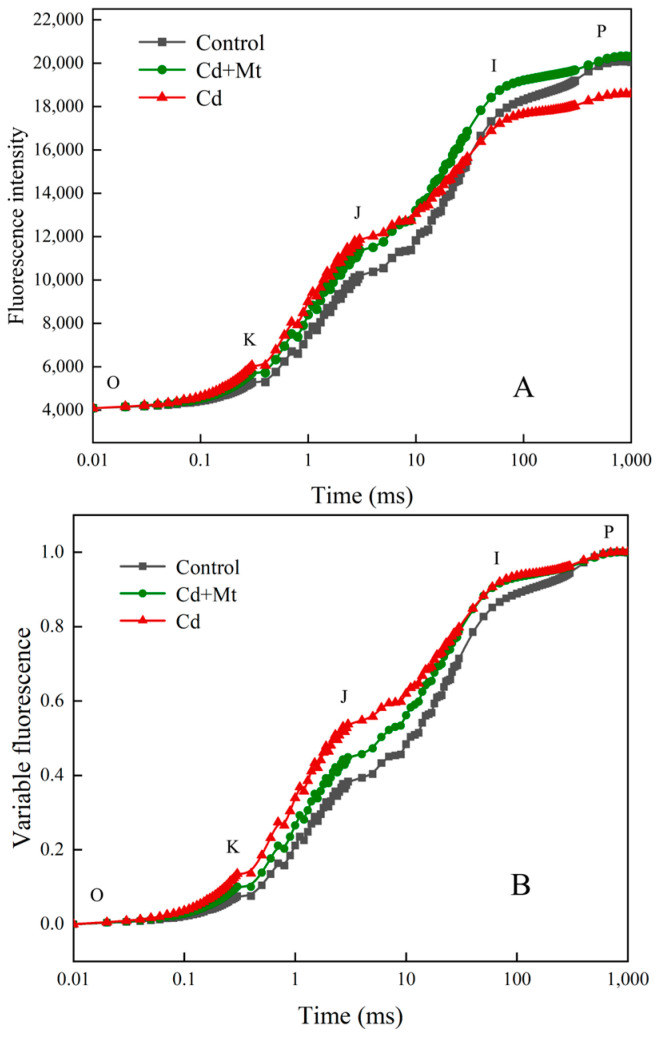
Chlorophyll fluorescence kinetics curves (**A**) and standardized chlorophyll fluorescence curves (**B**) for *R. simsii* leaves after 12 days under cadmium stress with the application of exogenous MT.

After 12 days of treatment, the OJIP fluorescence plots showed notable differences among the treatment groups (Figure 4A). Compared to the control treatment, both Cd and CdMT treatments exhibited a marked increase in fluorescence intensity at the J-point, with peak fluorescence (PF) in the Cd treatment slightly exceeding that of the CdMT treatment. In the following I-P phase, fluorescence intensity in the Cd treatment dropped more sharply, suggesting a more severe impact on the photochemical efficiency of PSII reaction centers in this group. Further normalization analysis (Figure 4B) indicated that variable fluorescence at the J-point increased by 19.8% and 47.0% in the Cd and CdMT treatments, respectively, compared to the control treatment. Notably, the J-point rise was more pronounced in the Cd treatment than in the CdMT treatment, while no significant differences were observed at the I-point among the treatments. These findings imply that Cd stress detrimentally affects PSII functionality, while exogenous MT application in the CdMT treatment appears to alleviate some of this negative impact, likely by supporting PSII stability and functionality under stress conditions.

When the donor side of PSII is damaged, chlorophyll fluorescence rises shortly after initiation (prior to the J-point), producing a distinct K-point, transforming the fluorescence profile from OJIP to OKJIP [26,27,28]. When analyzing OKJIP curves, a common approach is to normalize fluorescence changes from O to K (V_OK_) and from O to J (V_OJ_), calculated using the following equations:

V_OK_ = (F_T_ − F_O_)/(F_K_ − F_O_) (1)

V_OJ_ = (F_T_ − F_O_)/(F_J_ − F_O_) (2)

Here, F_O_ represents the minimum fluorescence in the dark-adapted state; F_J_ and F_K_ are fluorescence intensities at the J and K points, respectively; and F_T_ denotes the maximum fluorescence intensity during the measurement period. Normalization often reveals a clear K-band and L-band; an increase in fluorescence at 150–200 µs, indicated by a ΔL-band > 0, points to disrupted energy transfer due to basal thylakoid membrane dissociation.

**Figure 5:**
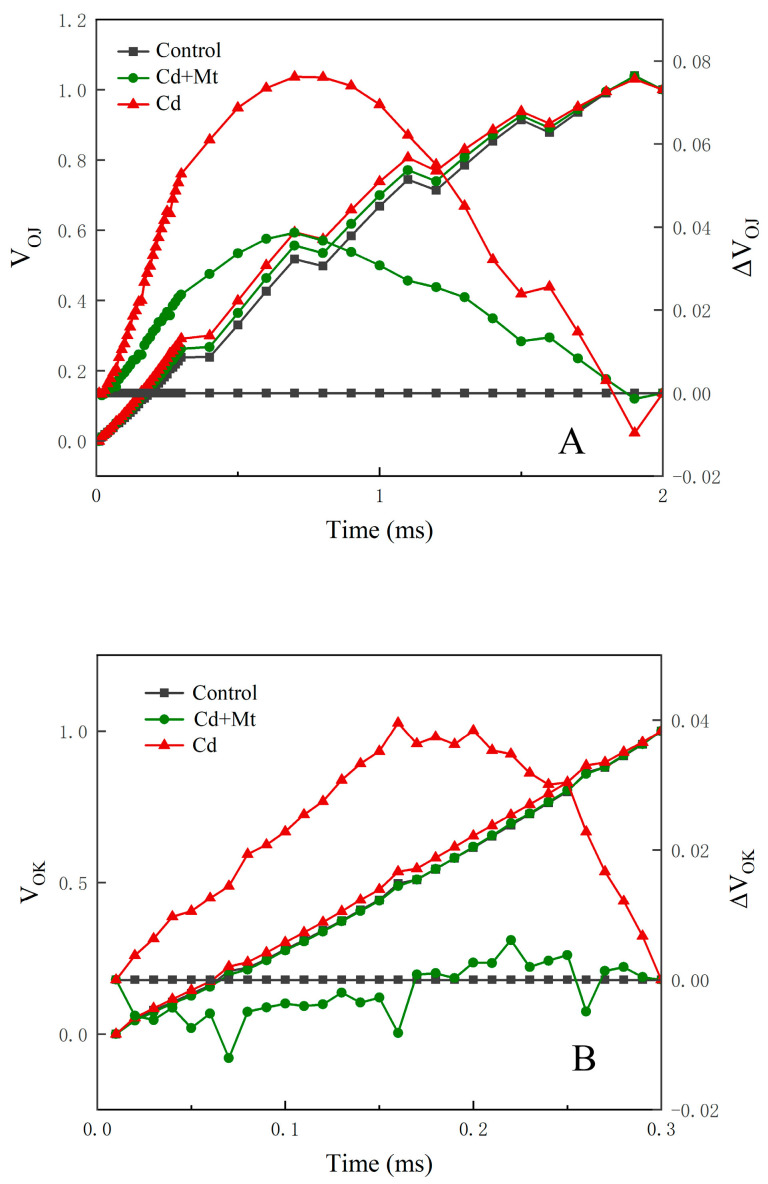
Effects of exogenous MT and cadmium stress on the K and L bands in *R. simsii* leaves for 12 days. Changes in the K-bands (**A**) and the L-bands (**B**) of *R. simsii* in response to Cd stress.

As illustrated in Figure 5A,B, the K and L bands in the Cd treatment group were markedly elevated compared to the control treatment group by day 12, signaling significant impairment in the electron transport chain due to Cd stress. Conversely, the CdMT treatment exhibited a smaller increase in the K-band, with the L-band closely resembling that of the control treatment. This suggests that exogenous MT application mitigates energy transfer disruptions in PSII, providing a protective effect on electron transport functionality under Cd stress conditions.

ΔV_OK_ = V_OK_(treatment) − V_OK_(control) (3)

ΔV_OJ_ = V_OJ_(treatment) − V_OJ_(control) (4)

### 2.4. Modulated Light Reflection Signals Measured at 820 nm

**Figure 6:**
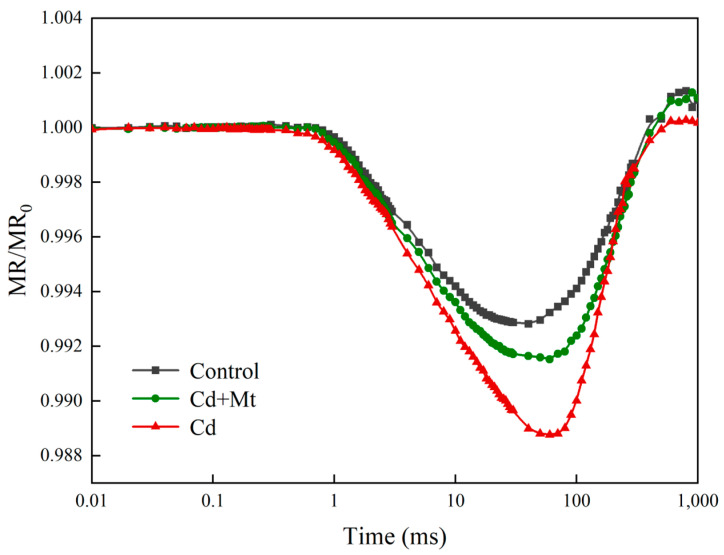
MR_820_ curves of *R. simsii* leaves under exogenous MT and cadmium stress for 12 days.

The findings from this study reveal significant alterations in the MR/MR_0_ kinetic curves of *R. simsii* leaves across different treatments. As illustrated in Figure 6, the MR/MR_0_ kinetic curve under CdMT treatment shows a delayed shift to its lowest point, which is both later and less pronounced compared to the control treatment by day 12. In contrast, under Cd treatment, the lowest point of the MR/MR_0_ kinetic curve occurs even later than in the CdMT treatment group, with a more substantial reduction at the lowest point. Additionally, the Cd treatment group displays the steepest maximum increase/decrease slope in the MR/MR_0_ curve. These observations suggest that exogenous MT application in the CdMT treatment moderates the stress on PSI reaction centers, reducing the extent of damage to the leaves. MT’s protective effect appears to mitigate the impact of Cd stress on the PSI electron transport chain, maintaining a more stable energy transfer process and thereby supporting the physiological resilience of *R. simsii* under Cd stress conditions.

### 2.5. Delayed Chlorophyll Fluorescence

The study of DF is an important tool for investigating plant photosynthesis, particularly in assessing responses to environmental stress. The DF signal at 20 µs not only reflects the shape dynamics of the induction curve over time but is also closely linked to the activity of P680, the primary chlorophyll molecule associated with the PSII reaction centers. DF intensity is directly influenced by the rate of back electron transfer (BET) in the PSII reaction centers, a critical process for maintaining PSII’s functional stability and homeostasis.

**Figure 7:**
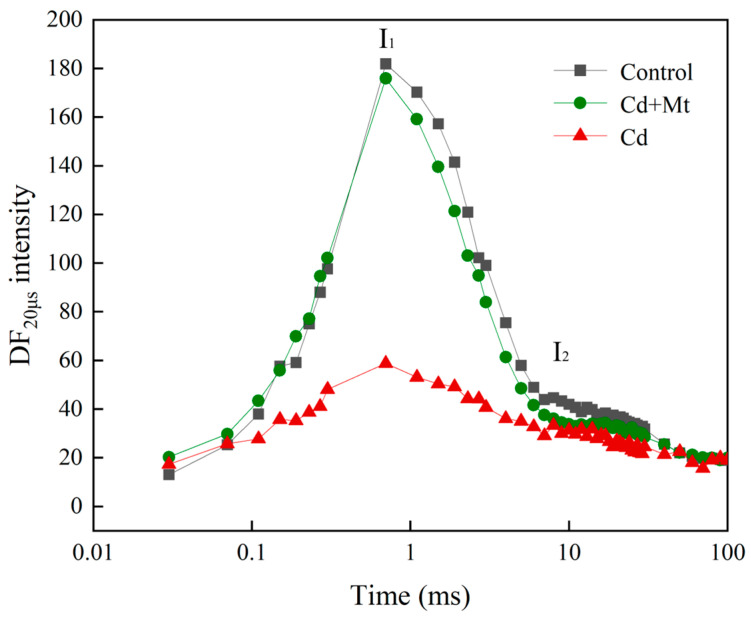
Delayed fluorescence induction curves of *R. simsii* leaves under exogenous MT and cadmium stress for 12 days. The peak at 3 ms was denoted as I_1_ and that at 100 ms was denoted as I_2._

As depicted in Figure 7, both I_1_ and I_2_ values in the Cd treatment group were significantly lower than those in the control treatment group, with a more substantial reduction observed in the I_1_ peak than in the I_2_ peak. This pattern suggests that under Cd stress, the electron donation capacity on the PSII donor side declines rapidly, impairing the reactivation of P680 and reducing the accumulation of oxidized and reduced states (Z^+^P680Q_a_Q_B_^−^ and Z^+^P680Q_a_^−^Q_B_) [29,30]. In comparison, the reduction in I_1_ and I_2_ values under CdMT treatment was less pronounced, indicating that exogenous MT helps to preserve PSII functionality to some degree, likely by stabilizing the electron transfer process within PSII. This protective effect of MT highlights its potential role in mitigating Cd-induced photodamage in *R. simsii*.

### 2.6. JIP Test

**Figure 8:**
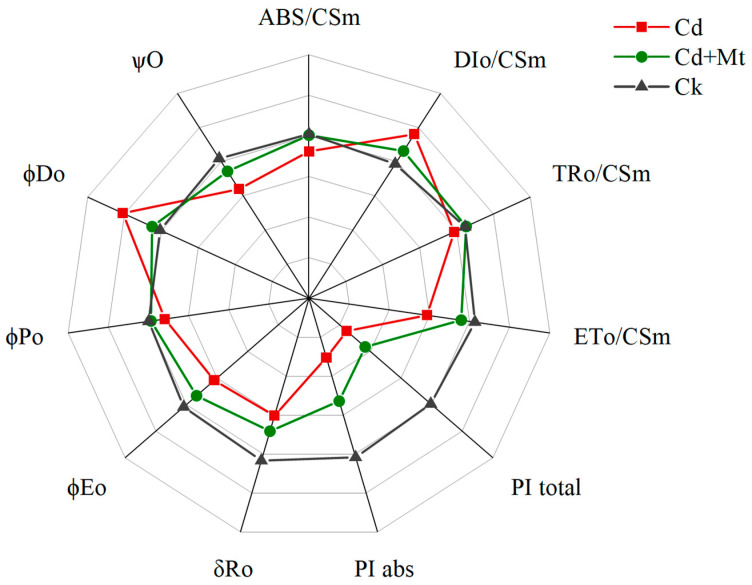
Radar plot of JIP test parameters of *R. simsii* leaves under exogenous MT and cadmium stress treatment for 12 days. For each parameter, the value for the comparison was set to 1.

The photosynthetic fluorescence characteristics of *R. simsii* leaves under different treatments were analyzed using the JIP test, a rapid quantitative method that interprets the kinetic chlorophyll fluorescence induction curves. This analysis provides insight into PSII light energy absorption, conversion, electron transport, and the functional status of PSII action centers, acceptors, and donor sides [31], thereby elucidating changes in the redox state of electron transporters. As depicted in Figure 8, the parameters ABS/CSm, TRO/CSm, ETO/CSm, and DIO/CSm were analyzed to evaluate the efficiency of each indicator per unit leaf cross-section at the point of peak fluorescence [32]. Under Cd stress, the parameters ABS/CSm, TRO/CSm, and ETO/CSm were significantly reduced, while DIO/CSm increased. Here, ABS/CSm represents the total energy absorbed per unit leaf area, TRO/CSm indicates the energy trapped by the active PSII reaction centers [33], and ETO/CSm reflects the electron transport flux per unit leaf cross-section. The observed decreases in these parameters suggest that Cd stress induces the excessive production of ROS, damaging the photosynthetic reaction centers and disrupting the pigment–protein complexes within the antennae. This leads to a decline in energy absorption and trapping efficiency on the leaf surface. DIO/CSm, representing thermal energy dissipation per unit leaf area, increased under Cd stress. This rise is attributed to a self-protective mechanism where the plant dissipates excess excitation energy as heat to prevent damage from retained energy, compensating for reduced electron transport efficiency. Notably, the increase in DIO/CSm was more pronounced in the Cd treatment, indicating more severe damage and a stronger self-protection response. Additionally, photosynthetic performance indicators PI_abs_ and PI_total_, representing overall photosynthetic efficiency, were significantly decreased under Cd stress. Key electron transport chain indicators φDO, φEO, and ΨO also showed declines. Parameters φEO and ΨO, which represent the efficiency of electron transport following Q_A_, primarily reflect PSII acceptor-side dynamics. The ΨO parameter also provides insights into recent center activity at 2 ms. Decreases in φEO and ΨO imply a reduced quantum yield from Q_A_ to PQ, reduced PSII recent center openness, and limited electron flow from Q_A_ to Q_B_. This reflects Cd’s damaging effect on PSII reaction centers, which disrupts electron transport on the acceptor side, ultimately leading to Q_A_^−^ accumulation—a phenomenon consistent with the observed OJIP curve shifts. Furthermore, φPo, representing maximum photochemical efficiency, decreased under Cd stress, indicating the worsening leaf stress. The parameter δRo, reflecting the efficiency of electron flow from the PSII electron transport chain to the terminal PSI electron acceptor, also declined, suggesting that Cd stress impairs PSI photochemical activity as well.

Although both the CdMT and Cd treatment groups exhibited similar parameter trends, the changes in the CdMT treatment were notably less severe. Macroscopically, the fluorescence characteristics and parameters in the CdMT treatment more closely resembled those of the control group, suggesting that exogenous MT mitigated Cd-induced damage to the leaves, reducing the extent of photoinhibition and helping to preserve PSII and PSI function.

### 2.7. Antioxidant Enzyme Activity and MDA Content

**Figure 9:**
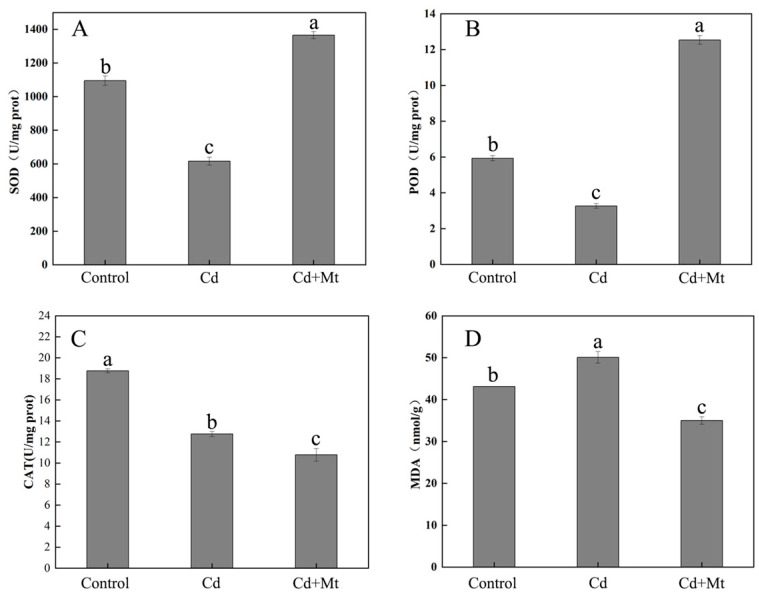
Changes in antioxidant enzyme activities and MDA content under various conditions. Different letters indicate significant differences determined by Duncan’s multiple range test at *p* ≤ 0.05. (**A**) Superoxide dismutase (SOD); (**B**) peroxidase (POD); (**C**) catalase (CAT); (**D**) malondialdehyde (MDA).

*R. simsii* subjected to Cd stress exhibited a significant reduction in SOD and POD activities compared to the control group, indicating compromised antioxidant defense under Cd stress. However, MT treatment markedly increased SOD and POD activities (Figure 9A,B), highlighting its role in stimulating the antioxidant enzyme system. Furthermore, the decreased leaf MDA content in the MT treatment group reflects reduced lipid peroxidation and oxidative damage (Figure 9D). Interestingly, CAT activity showed a slight decline after melatonin application under Cd stress (Figure 9C), suggesting the differential regulation of antioxidant enzymes. Overall, MT enhanced the antioxidant defense system, mitigated Cd-induced oxidative stress, and improved the Cd stress tolerance of *R. simsii.*

## 3. Discussion

In this study, the photosynthetic physiological parameters of *R. simsii* leaves under Cd stress were meticulously analyzed using the LI-6400 portable photosynthesis system (Figure 2). The results indicate that Cd stress significantly suppressed Pn, Gs, and Ci in *R. simsii* leaves. The reduction in stomatal conductance and intercellular CO_2_ suggests that stomatal closure may be the direct factor leading to decreased photosynthesis. Cd is known to influence stomatal dynamics through various mechanisms, such as inducing ultrastructural changes in guard cells, mimicking calcium ion activity that triggers potassium ion efflux, and disrupting the synthesis and transport of phytohormones (including auxins and cytokinins), which ultimately affects mesophyll conductance and cell membrane function [34,35].

Under Cd stress, the stomata of *R. simsii* leaves exhibited significant impairment, as evidenced by reduced stomatal conductance and closure. However, the application of exogenous MT notably enhanced both the net photosynthetic rate and stomatal conductance in *R. simsii* leaves, suggesting MT’s protective and regulatory role in maintaining stomatal function. To support this, a study by Erland Studio used quantum dot nanoparticles to visualize MT in stomatal structures, finding predominant localization in guard cells [36]. Similarly, research by Mir studio on mustard plants showed that MT application increased stomatal aperture size [37]. MT pretreatment has also been shown to enhance traits such as stomatal density, length, and width, likely by regulating stomatal movement through hormonal and other signaling pathways. This regulatory effect on stomatal structure and function could explain MT’s role in reducing Cd-induced damage and improving photosynthetic efficiency under heavy metal stress conditions.

The analysis of photosynthetic fluorescence parameters revealed significant fluctuations in the OJIP curves of *R. simsii* leaves under prolonged Cd stress. As shown in Figure 4, both Cd treatment and CdMT treatment groups displayed an increase in Fo compared to the control, with all Fo values aligned at the O-point for comparison. The rise in Fo likely reflects reversible inactivation or irreversible damage to the PSII reaction centers or thylakoid membrane damage [38], a response also observed under drought stress conditions. The OJIP fluorescence curve consists of three phases—O-J, J-I, and I-P—which correspond to distinct electron transport processes [39]. The O-J segment represents the initial light-induced electron capture via the PSII antenna pigment–protein complex, leading to electron transfer via pheophytin (Pheo) to the primary quinone receptor Q_A_ and creating P^+^PheoQ_A_^−^ radicals [40]. The J-point marks equilibrium between oxidized (Q_A_^+^) and reduced (Q_A_^−^) states, while the J-I segment represents electron transfer from Q_A_^−^ to Q_B_. Although PSI emits weak fluorescence, the I-P phase in the OJIP curve closely reflects electron transfer interactions between PSII and PSI [41]. The I-point denotes equilibrium in the PQ pool’s reduction and re-oxidation in PSII, while the P-point marks the full reduction of electron acceptors in PSII and PSI [42].

In Figure 3A, a notable decrease in the I-P region was observed on day 12 relative to day 0, suggesting compromised PSII-PSI electron transfer. After normalization (Figure 3B), the J-point initially declined before rising with prolonged Cd stress, reaching its peak on day 12. The initial J-point downregulation indicates a stress-induced activation of photosynthetic defense mechanisms. Conversely, the later J-point increase implies that electron capture via P680 in the PSII reaction centers surpassed the Q_A_-PQ electron exchange rate at the Q_B_ site, leading to Q_A_^−^ accumulation and reduced electron transfer efficiency. This imbalance likely results from Cd-induced constraints on PSII’s donor and acceptor sides or hindered the uptake of electron-carrying elements like iron and manganese. The P-point gradually decreased with extended Cd exposure, likely due to a reduction in PSII antenna pigments, an increase in non-photochemical quenching, and a reduction in active PSII reaction centers [43]. The CdMT treatment demonstrated a smaller J-point increase compared to the Cd treatment alone, indicating enhanced electron transfer efficiency from Q_A_^−^ to Q_B_ and greater electron acceptance capacity in the PQ pool.

Additionally, a prominent K-point (Figure 4) emerged under Cd treatment, likely due to Cd^2+^ competing for the Ca^2+^ binding site on the Mn_4_Ca cluster in the PSII electron donor photosynthetic oxygen-evolving complex (OEC). This competition results in a decrease in the photosynthetic oxygen release rate, as the substrate H_2_O cannot effectively supply the electrons required for the efficient operation of PSII [44,45]. The reduced K-band (Figure 5A) in the CdMT treatment implies that MT may play a protective or reparative role within the OEC. The presence of an L-band indicates weakened electron acceptance via the PQ pool, blocking electron transfer from Q_A_^−^ to Q_B_ and contributing to the elevated J-point. The L-band (Figure 5B) serves as an indicator of the connectivity between PSII reaction centers and antenna proteins; reduced connectivity signifies heightened damage. Enhanced connectivity between PSII reaction centers and antennae proteins observed under MT treatment suggests that exogenous MT mitigates Cd-induced damage, supporting better PSII functionality under heavy metal stress.

In the photochemical reactions of photosynthesis, the reversibility of charge separation and electron transport processes is essential for maintaining PSII functionality. During the primary photochemical reaction, light energy excites the PSII reaction center P680 to a high-energy state (P680*), initiating electron transfer through a series of acceptors, which ultimately leads to water oxidation and oxygen release [46]. Charge recombination following separation can occur during the formation and decay of P680, a process critical for PSII recycling. Under specific conditions, P680 can transfer its excitation energy back to the antenna pigment, resulting in DF, which is closely linked to the redox state of P680 [46,47].

On the DF curve, characteristic points I_1_ and I_2_ correspond to distinct physiological processes. The appearance of the I_1_ point signifies the accumulation of the luminescent complex Z^+^P680QₐQ_B_^−^ and the establishment of transmembrane potential induced by PSI oxidation. Conversely, the I_2_ point correlates with the PQ pool’s reduced state, indicating the accumulation of Z^+^P680Qₐ^−^Q_B_^−^ [48]. Decreased I_1_ and I_2_ points under Cd stress (Figure 7) indicate reduced electron donation to P680, decreased reaction center activity, and inhibited P680 reactivation, limiting light-harvesting complex formation. This response may be due to a decrease in OEC efficiency, consistent with observations in rapid fluorescence induction curves. Although DF and transient fluorescence derive from distinct mechanisms, both serve as indicators of photosynthetic electron transport chain status. Transient fluorescence highlights the reduction state of the primary electron acceptor Q_A_^−^ at various time points, while DF occurs during charge recombination in different PSII states. Consequently, DF provides an intuitive reflection of PSII’s functional state. Enhanced DF intensity indicates an accelerated BET rate in PSII, likely associated with OEC protection and the regulatory effects of exogenous MT on the photosynthetic electron transport chain.

In PSI, the primary electron donor, P700, can absorb light at 820 nm in its oxidized form, P700^+^ [49,50]. Thus, changes in light absorption or reflection can serve as indicators of the redox state of P700 and PC in PSI. Modulated reflectance measurements at 820 nm complement transient fluorescence analysis by providing insights into electron flow rates from PSII to PSI [51,52]. Under Cd stress, PSI and PC oxidation are affected (Figure 6), as reflected by an increase in the magnitude and speed of the MR_820_ curve’s decrease. However, reliance solely on the 820 nm reflectance kinetic curve has limitations since Cd stress may disrupt PSI-PSII coordination. Therefore, simultaneous measurements of transient fluorescence and 820 nm reflectance were conducted using the M-PEA instrument. As observed, the steepest decline in the MR_820_ curve corresponds to the O-J segment of the OJIP curve. When the MR_820_ curve reaches its lowest point, usually aligning with the J-I segment, it represents equilibrium in the redox state of the PQ pool. At this stage, electron provision via the PQ pool to PC^+^ and P700^+^ matches the electron transport rate from PC and P700 to their acceptor sites. Under Cd stress, the MR_820_ curve’s lowest point was significantly delayed, indicating prolonged PQ pool equilibration, reflecting Cd’s impact on redox homeostasis and electron chain transfer efficiency.

The study by Dr. Wodala shows that Cd stress can impair the photochemical efficiency of PSI and decrease the number of electrons in the electron transport chain between the photosystems [53]. Furthermore, ROS generated in the thylakoid membrane under Cd stress can directly damage the Fe-S center of ferredoxin in PSI, thereby reducing stromal NADPH production in the thylakoids [54]. In contrast, the CdMT treatment leaves reached the MR_820_ lowest point earlier, suggesting that MT’s antioxidant properties mitigate Cd stress and the support recovery of the plant’s internal physiological state. In previous studies [22], we concluded that exogenous MT treatment can increase the expression level of *RhPGR5A* in the heat-stressed rhododendron, thereby enhancing PGR5-mediated cyclic electron flow (CEF), which reduces the adverse effects on PSI activity and overall electron transport rates (ETRs). Although the induction mechanisms of heat stress and Cd stress differ, we hypothesize that MT similarly regulates CEF by modulating gene expression, thereby influencing photosynthetic electron transport. Moreover, as an effective antioxidant, MT can scavenge excess ROS. Consequently, leaves treated with CdMT reached the lowest point of the MR_820_ curve more rapidly, indicating that MT facilitates a faster recovery of the PQ pool to a steady state and alleviates the impact of Cd stress on the plant’s redox system. Cd stress elevates intracellular ROS, while MT, as a potent antioxidant, scavenges excess ROS. As shown in Figure 9, MT significantly enhanced the activities of SOD and POD in *R. simsii* under Cd stress, thereby improving the plant’s ability to scavenge superoxide anions and maintain redox homeostasis, helping maintain redox balance. Consequently, CdMT-treated leaves reach the MR_820_ curve’s lowest point sooner, indicating that MT aids in faster PQ pool homeostasis, alleviating Cd stress effects on the plant’s redox system.

## 4. Materials and Methods

### 4.1. Plant Material and Handling

This research was conducted in December 2023 within a controlled incubator environment in the laboratory building of Jiyang College, Zhejiang Agriculture and Forestry University, located in Zhuji City, Zhejiang Province, China. The study utilized *R. simsii* seedlings, cultivated from one-year-old cuttings with uniform growth characteristics, which were supplied by Zhejiang Shanjuan Horticulture Co., based in Jiaxing City, Zhejiang Province, China. Initially, the seedlings were grown in a substrate comprising a volumetric mix of vermiculite, perlite, and peat soil in a 1:1:3 ratio. Following a one-week acclimation period in the growth chamber, the seedlings’ root systems were carefully rinsed and transferred into a 1/4-strength Hoagland nutrient solution. This nutrient solution contained 1.0 mM calcium nitrate (Ca(NO_3_)_2_), 1.0 mM potassium nitrate (KNO_3_), 0.4 mM magnesium sulfate (MgSO_4_), 0.2 mM ammonium dihydrogen phosphate ((NH_4_)H_2_PO_4_), 20 μM sodium ferric ethylenediaminetetraacetic acid (NaFeEDTA), 0.5 μM manganese chloride (MnCl_2_), 3.0 μM boric acid (H_3_BO_3_), 0.4 μM zinc sulfate (ZnSO_4_), 1 μM ammonium molybdate ((NH_3_)_6_Mo_7_O_24_), and 0.2 μM copper sulfate (CuSO_4_). The nutrient solution was adjusted to a pH of 6.0 to optimize nutrient availability and stability. The experimental setup included three treatment groups, control treatment, cadmium (Cd) treatment, and cadmium with melatonin (CdMT) treatment, each comprising six biological replicates. After a week of acclimatization and initial growth, the nutrient solution was renewed to maintain consistency. Incubator conditions were set to simulate diurnal variation, with daytime temperatures of 24 ± 2 °C and night-time temperatures of 16 ± 2 °C, under a 16 h light/8 h dark photoperiod. Light intensity ranged from 500 µmol m^−2^ s^−1^ to 600 µmol m^−2^ s^−1^ photosynthetic photon flux density, and relative humidity was maintained at approximately 60%.

For the Cd treatment group and CdMT treatment group, a 400 μmol/L solution of cadmium chloride (CdCl_2_) was introduced to the culture medium. In the CdMT treatment group, a 200 μmol/L MT solution was applied to the seedlings at 7:30 p.m., beginning from the top leaves to the base of the plant. The melatonin solution, containing 200 µM melatonin dissolved in water, was stored in a 150 mL black-shaded spray bottle and applied in full volume for each treatment. During the MT application, ambient light was minimized to prevent MT degradation caused by exposure to light. MT applications were performed every two days. To ensure experimental consistency across treatments, ultrapure water was sprayed on the seedlings in the control treatment group at the same time intervals as the Cd treatment group. Photosynthetic physiological parameters of seedlings across all treatments were systematically recorded every three days, enabling an in-depth analysis of MT’s impact on photosynthesis under Cd stress.

### 4.2. Gas Exchange Measurements

In this study, comprehensive gas exchange parameters of *R. simsii* seedling leaves were measured using a portable photosynthetic gas exchange measurement system-Model LI-6400 (LI-COR, Lincoln, NE, USA). To ensure data accuracy, representativeness, and comparability, measurements were conducted on the third leaf pair from the apex of each seedling’s stem. The system’s integrated red and blue LED light sources provided a stable photosynthetic photon flux density (PPFD) of 1200 µmol m^−2^ s^−1^, with ambient CO_2_ levels maintained at 400 µmol mol^−1^ to simulate field conditions. The leaf chamber was precisely regulated to 25 °C, with an airflow rate of 300 mmol s^−1^, replicating natural wind conditions to accurately assess the gas exchange dynamics under controlled environmental parameters.

### 4.3. Simultaneous Measurement of PF, DF, and MR Kinetics

In this study, we selected the third pair of mature and functionally intact leaves at the top of the plant stem as the experimental material. To ensure the accuracy of chlorophyll fluorescence parameter measurements, these leaves were dark-adapted under light-free conditions for 30 min prior to fluorescence determination. Quantitative analysis of chlorophyll fluorescence was carried out with an M-PEA (Hannsatech Instruments Ltd., King’s Lynn, UK) fluorescence measurement system, which has the ability to measure fluorescence parameters with high accuracy. During the fluorescence induction phase, a 650 nm red light pulse at an intensity of 3500 µmol m^−2^s^−1^ was applied to optimize the excitation efficiency of the fluorescence signal. In analyzing the fluorescence response curve, point O represented the initial fluorescence level, while points K (0.3 ms), J (2–3 ms), and I (30 ms) corresponded to specific critical time points in the fluorescence rise curve. Point P (500 ms) indicated the steady-state peak of the chlorophyll fluorescence curve.

In addition, the JIP test, based on chlorophyll fluorescence kinetics, was employed to quantitatively characterize photosynthetic parameters within the OJIP transient [55]. This test allowed for the evaluation of absorbed excitation energy (ABS), energy capture for trapping (TR), electron transport (ET), and energy dissipation (DI), thus enabling a detailed understanding of energy flow through the photosynthetic membrane system and the calculation of energy flux ratios. To analyze the transient fluorescence kinetics of PSII, the following parameters were measured: minimum fluorescence (F_O_) at 20 μs, K-point fluorescence (F_K_) at 0.3 ms, J-point fluorescence (F_J_) at 2–3 ms, I-point fluorescence (F_I_) at 30 ms, and maximum fluorescence (F_P_) [56]. The maximum quantum efficiency of PSII was calculated with the following equation:

(Fᵥ/F_M_) = (F_M_ − F_O_)/F_M_ (5)

Variable fluorescence parameters V_OK_ and V_OJ_ were calculated with the following equations:

V_OK_ = (F_T_ − F_O_)/(F_K_ − F_O_) (6)

V_OJ_ = (F_T_ − F_O_)/(F_J_ − F_O_) (7)

See Reference [57] for more information.

For the 820 nm light reflectance kinetics, we employed the ratio MR/MR_0_, where MR_0_ is the reflectance value at the initial illumination (0.7 ms), and MR represents the reflectance of 820 nm light during subsequent illumination [58]. In DF kinetics, I_1_ and I_2_ correspond to DF signals recorded at 7 ms and 200 ms, respectively. The DF decay kinetics curves were fitted to the following equation:

DF(t) = L_1_ × exp(−t/τ_1_) + L_2_ × exp(−t/τ_2_) + L_3_ (8)

where L_1_, L_2_, and L_3_ denote the amplitudes of the DF components, and τ_1_ and τ_2_ represent their respective decay constants.

The accuracy and reproducibility of the experimental data should be ensured when performing such analyses. In addition, possible physiological and biochemical explanations for the observed changes in fluorescence and light reflectance should be considered, as well as their implications for the photosynthetic mechanism.

### 4.4. Antioxidant Enzyme Activity Assay and Determination of Malondialdehyde

The activities of antioxidant enzymes, including superoxide dismutase (SOD), peroxidase (POD), and catalase (CAT), and the malondialdehyde (MDA) content were determined using commercial kits (Comin, Suzhou, China), following the manufacturer’s protocols.

### 4.5. Data Analysis

In this study, the experimental data were presented in the form of mean values followed by standard errors, and statistical analysis was performed using SPSS 20.0 software (IBM, Armonk, NY, USA). In order to assess the significant differences between the different treatment groups, the least significant difference (LSD) test was used, and the difference between the two groups was considered statistically significant when the *p*-value < 0.05.

## 5. Conclusions

This study demonstrated the beneficial impact of exogenous MT on the photosynthetic performance of *R. simsii* leaves subjected to Cd stress by comparing gas exchange parameters, chlorophyll fluorescence characteristics, antioxidant enzyme activity, and 820 nm modulated reflectance among the control, Cd, and CdMT treatment groups. Cd stress significantly reduced the net photosynthetic rate, stomatal conductance, and intercellular CO_2_ concentration in leaves. However, exogenous MT application mitigated these inhibitory effects, showing a protective regulatory effect on stomatal function.

Moreover, MT not only partially restored PSII functionality but also reestablished ionic balance around PSII, enhanced the reductive kinetics of PSI reaction centers and PC, and shortened the time required for the PQ pool to reach equilibrium. DF analysis further confirmed that MT enhanced the reactivation efficiency of P680 and protected the OEC from Cd-induced damage. Additionally, exogenous MT significantly increased the activities of antioxidant enzymes, including POD and SOD, while markedly reducing MDA content, thereby mitigating oxidative damage and enhancing cellular stability under Cd stress.

In conclusion, exogenous MT significantly enhanced Cd stress tolerance in *R. simsii* by protecting the photosynthetic machinery, boosting antioxidant defenses, maintaining redox homeostasis, and regulating stomatal function. These findings highlight the potential of MT as a practical and effective plant growth regulator for mitigating heavy metal stress in plants. Importantly, this study provides a scientific basis for developing novel strategies to enhance crop resilience in Cd-contaminated soils, which is of great significance for sustainable agriculture and environmental management. Furthermore, it offers new insights into the molecular and physiological mechanisms of plant photosynthetic responses under heavy metal stress, paving the way for future research into stress tolerance mechanisms and MT applications in other plant species.

## Data Availability

The data presented in this study are available on request from the corresponding author. The data are not publicly available due to privacy reasons.
